# Does ozone exposure affect herbivore-induced plant volatile emissions differently in wild and cultivated plants?

**DOI:** 10.1007/s11356-020-09320-z

**Published:** 2020-05-28

**Authors:** Agnès Brosset, Amélie Saunier, Minna Kivimäenpää, James D. Blande

**Affiliations:** Department of Environmental and Biological Sciences, University of Eastern Finland, FIN-70211 Kuopio, Finland

**Keywords:** *Brassica*, *Sinapis*, *Raphanus*, Volatile organic compound, Herbivore-induced plant volatile, Terpenes, Ozone

## Abstract

Concentrations of tropospheric ozone have more than doubled in the Northern Hemisphere since pre-industrial times. Plant responses to single abiotic or biotic stresses, such as ozone exposure and herbivore-feeding, have received substantial attention, especially for cultivated plants. Modern cultivated plants have been subjected to selective breeding that has altered plant chemical defences. To understand how ozone might affect plant responses to herbivore-feeding in wild and cultivated plants, we studied the volatile emissions of brassicaceous plants after exposure to ambient (~ 15 ppb) or elevated ozone (80 ppb), with and without *Plutella xylostella* larvae-feeding. Results indicated that most of the wild and cultivated plants increased volatile emissions in response to herbivore-feeding. Ozone alone had a weaker and less consistent effect on volatile emissions, but appeared to have a greater effect on wild plants than cultivated plants. This study highlights that closely related species of the *Brassicaceae* have variable responses to ozone and herbivore-feeding stresses and indicates that the effect of ozone may be stronger in wild than cultivated plants. Further studies should investigate the mechanisms by which elevated ozone modulates plant volatile emissions in conjunction with biotic stressors.

**Electronic supplementary material:**

The online version of this article (10.1007/s11356-020-09320-z) contains supplementary material, which is available to authorized users.

## Introduction

Since pre-industrial times, concentrations of gaseous pollutants have increased in the Northern Hemisphere (Mills et al. 2018; Volz and Kley 1988). These pollutants affect air quality and have an impact on the climate, through the effects of gases with a positive radiative forcing (Scott et al. 2014). For instance, nitrogen oxides (NO_x_), mostly released as products of fossil fuel combustion, are involved in ozone formation in the troposphere through a series of reactions also involving volatile organic compounds (VOCs) (Atkinson and Arey 2003). Exposure of plants to ozone causes foliar damage due to the induction of oxidative stress in plant cells (Baêsso et al. 2018; Vainonen and Kangasjärvi 2015). Ozone exposure can affect carbon assimilation through the disruption of stomatal control and limit photosynthesis that is essential for plant growth and defence (Black et al. 2007; Fiscus et al. 2005; Morgan et al. 2003).

The *Brassicaceae* is one of the largest families of annual and biannual plants; it is widely diversified, consisting of oil and vegetable crops, and is globally distributed, cultivated and consumed (Gómez-Campo and Prakash 1999). A focus on rearing plants to have high yield, strong growth performance and good taste has often been at the expense of plant defence against herbivores (Chaudhary 2013). In the *Brassicaceae*, glucosinolate content has been reduced to improve taste and reduce carcinogenic risk (Mithen et al. 1987). However, glucosinolates and their reaction products also provide protection against generalist and specialist insects such as larvae of *Mamestra brassicae* (Gols et al. 2008) and *Pieris rapae* (Jeschke et al. 2017; Agrawal and Kurashige 2003). Selection procedures have resulted in modern cultivated plants being morphologically and chemically distant from their wild relatives. Differences in the defence properties of cultivated plant varieties and their wild relatives have been assessed in several species, for example maize (*Zea mays*) and its wild ancestor *Balsas teosinte* (Maag et al. 2015; De Lange et al. 2014) and cabbage *Brassica oleracea* (Gols et al. 2008). In both species, studies reported a reduction in the VOCs emitted in response to herbivory as well as a reduction in the resistance to herbivory of cultivated plants compared to their wild relatives. In a changing world with an increase in atmospheric pollutants, it has become a priority to understand how ozone might affect plant responses to herbivore-feeding in a wide range of plant species, including wild and cultivated plants.

Several studies have shown that ozone stress can affect plant responses to herbivore-feeding (Blande et al. 2014). Elevated ozone applied at a concentration of 100 ppb decreased emissions of several terpenes emitted by oilseed rape (*Brassica napus* ssp. *oleifera*) in response to *Plutella xylostella* feeding (Himanen et al. 2009). In contrast, *Brassica nigra* emitted greater quantities of herbivore-induced volatiles in response to *Pieris brassicae*-feeding under 120-ppb ozone than under ambient ozone (Khaling et al. 2016). These results show that interactions between ozone and herbivore-feeding stresses might act in synergistic or antagonistic ways, inducing higher or lower emissions of herbivore-induced plant volatiles (HIPVs). Simultaneous or sequential exposures to ozone and herbivore-feeding have the potential to induce volatile blends that are quantitatively and qualitatively different to those emitted by plants exposed to ozone or herbivore-feeding stresses separately (Becker et al. 2015). Such changes in plant volatiles emitted in response to herbivory could influence volatile-mediated interactions. For instance, *B. nigra* plants exposed to *P. brassicae*-feeding under 120-ppb ozone attracted more parasitoid wasps (*Cotesia glomerata*) in oriented flight choice tests than plants exposed to herbivore-feeding under ambient ozone conditions (Khaling et al. 2016).

In this study, we utilised a range of wild and cultivated *Brassicaceae* species to investigate the effects of elevated ozone on the VOCs emitted in response to herbivory. We exposed plants to either ambient or elevated ozone (80 ppb) for 7 days. Half of the plants grown under each ozone condition were infested with *Plutella xylostella* larvae after 5 days of the starting ozone exposure and allowed to feed for 2 days (Online Resource 1). Eight *Brassicaceae* species, *Barbarea vulgaris*, *Brassica nigra*, *Brassica napus oleifera*, *Brassica juncea*, *Sinapis arvensis*, *Sinapis alba*, *Raphanus raphanistrum*, and *Raphanus sativus*, were utilised in the study. We hypothesised that herbivore-feeding would induce volatile emissions in all plants, but that elevated ozone exposure would modulate the volatile emissions induced by herbivory in the wild and cultivated *Brassicaceae* tested.

## Material and methods

### Plant growth and insect rearing

This study utilised eight species of the *Brassicaceae*, including four wild and four cultivated varieties. Of the wild varieties, *Brassica nigra* (black mustard), *Sinapis arvensis* (charlock mustard) and *Raphanus raphanistrum* (wild radish) were obtained from wild populations in the Netherlands (supplied by E. Poelman of Wageningen University), and *Barbarea vulgaris* (yellow rocket) was obtained from a wild population in Kuopio, Finland. The four cultivated varieties utilised were *Brassica napus oleifera* var. Hobson (forage rape, Kings, UK), *Brassica juncea* var. Vitasso (brown mustard, Kings, Italy), *Sinapis alba* (mustard, seeds provided by E. Poelman of Wageningen University, the Netherlands) and *Raphanus sativus* (radish, Nojaus seklos, Lithuania). Plant seeds were sown individually in 0.8-L plastic pots containing a mix of peat, soil and sand (3:1:1). The plants were grown in controlled environment chambers (Weiss Technik, Lindenstruth, Germany) with an artificial light-dark cycle (16L:8D), PAR approximately 350 μmol m^−2^ s^−1^, day and night temperatures equal to 21 °C and 16 °C, respectively, and day and night relative humidity equal to 60.0% and 80.0%, respectively. The plants were watered daily and fertilised twice per week (0.1% solution, N:P:K, 19:4:20 (Kekkilä Oyj, Finland)).

The diamondback moth, *Plutella xylostella*, is a specialist *Brassica*-feeding herbivore. Moths were reared in an insectary on broccoli (*Brassica oleracea* var*.* Italica) with an artificial light-dark cycle (16L: 8D) at 23 ± 0.5 °C.

### Experimental design

Four-week-old plants were divided into four groups and moved into four separate chambers (Online Resource 1). Three to five replicates were tested per treatment (*n* are reported in the Online Resource 2). The treatments in the four chambers were (AB) ambient ozone (15–20 ppb); (HB) 48 h of herbivore-feeding/ambient ozone (15–20 ppb); (O_3_) elevated ozone (80 ppb); and (O_3_+HB) 48 h of herbivore-feeding/elevated ozone (80 ppb). The ambient ozone level in our laboratory chamber room was used as the background control level and was measured to be approximately 15 ppb. A concentration of 80 ppb for 5 days was used to mimic a period of high daily ozone levels (Vingarzan 2004). In the elevated ozone treatments, the ozone level was raised to 80 ppb at 7:00 and then lowered to 30 ppb at 20:00. We used this method to follow a realistic daily oscillation in ozone concentration. Ozone was generated in the chambers with an ozone generator (OZ 500 ozone generator, Fisher, Germany) and continuously monitored with an ozone analyser (model ozone 42M, Environnement S.A., France). Exposure to ambient or elevated ozone was continued for 5 days (Online Resource 1). On the fifth day, all plants in the HB and O_3_+HB chambers were infested with 12 second and third instar *P. xylostella* larvae for 48 h. After 48 h of feeding—7 days from the initiation of ozone exposure—we collected volatile emissions from plants. We rotated chambers between the four treatments. Different species were tested at different times to avoid volatile contamination altering the profiles that would be collected.

### Volatile organic compound collection

Volatile organic compounds were collected using the process of dynamic headspace sampling. Plants were enclosed in plastic bags (polyethylene terephthalate; overall dimensions 35 × 43 cm; Look® Isopussi Eskimo oy, Finland) that were previously baked for 1 h at 120 °C. Air filtered through activated charcoal was pumped into bags at 300 mL min^−1^ until they had been fully flushed. Two lamps (model Shuttle Plus, LIVAL, Sipoo, Finland) were placed over the plants (PAR, approximately 350 μmol m^−2^ s^−1^) to maintain similar light to that in the chambers. Volatiles were collected in stainless steel tubes filled with 200 mg Tenax TA 60/80 adsorbent (Markes International Ltd., UK) with a flux of 0.22 L min^−1^ using a vacuum pump (KNF, Germany). For each sampling round, a blank (empty bag) sample was taken using a similar method. The collection time was 1 h.

### Volatile compound analysis

The samples were analysed by gas chromatography–mass spectrometry (Hewlett-Packard GC type 6890, Germany; MSD 5973, UK). Trapped compounds were desorbed with an automated thermal desorption unit (PerkinElmer ATD400 Automatic Thermal Desorption System, Wellesley, USA) at 250 °C for 10 min and cryofocused at − 30 °C. The compounds were then transferred in a split mode 1:20 to an HP-5MS capillary column (0.25 μm × 60 m × 0.25 μm, Agilent Technologies, USA). The carrier gas was helium. The oven temperature was held at 40 °C for 2 min and then programmed to ramp by 5 °C min^−1^ to 210 °C and then by 20 °C min^−1^ to 250 °C under a constant flow of 1.2 mL min^−1^. Mass spectra were acquired by scanning from 33 to 400 *m*/*z*. Compound identification was made by comparison with the injection of 30 analytical standards (Sigma-Aldrich, USA). For the unknown compounds, we calculated the retention indices RI, through the injection of alkanes C8-C20 and compared their mass spectra to those in the NIST and Wiley libraries. Compound quantification was based on using the total ion chromatograms (TIC) and according to the responses of analytical standards. Volatile organic compound emission rates (*E*) were calculated by considering the compound amounts in the inlet and outlet air as follows: $E=\frac{F\times \left(C2-C1\right)}{m},$ where *E* is the emission rate expressed in ng g^−1^ h^−1^, *F* is the flow rate to the bag (L min^−1^), *C*1 is the mass of volatile compound in the incoming air (ng), *C*2 is the mass in the outgoing air (ng) and *m* is the dry biomasses of plants (g) obtained after 3 days of drying in an oven at 60 °C.

### Statistical analyses

Statistical analyses were performed using SPSS 25 software (IBM Corp. Armonk, USA). First, the emissions of all compounds were summed for each plant species and then compounds were grouped as total volatile emissions, green leaf volatiles (GLVs, including methyl salicylate (MeSA)), monoterpenes (including the homoterpene (*E*)-4,8-dimethyl-1,3,7-nonatriene ((*E*)-DMNT)), sesquiterpenes and nitrogen- and sulphur-containing compounds (N-, and S-containing compounds). We checked the normality of the data with a Shapiro-Wilk test and the homogeneity of the variance with a Levene test. In cases that these assumptions were not fulfilled, logarithmic or square root transformations were conducted. We assessed the ozone, herbivory and plant type (wild or cultivated) effects on emission rates, with linear mixed models ANOVA. Ozone, herbivory and plant type were the fixed factors and species the random factor. Ozone and herbivory effects on volatile emissions of each species were assessed with two-way ANOVA.

The effects of the treatments on emissions of the individual volatile compounds were also tested. The emission rates data did not meet the assumptions of normality after transformation; therefore, generalised linear models (GLMs) were used to test the main and interaction effects of ozone and herbivory. Due to the large number of volatile compounds emitted in the eight plants studied, we decided to do partial least squares–discriminant analysis (PLS-DA) to determine the compounds most representative of the variance (Online Resources 3 and 4). Metaboanalyst (https://www.metaboanalyst.ca/) was used to generate the PLS-DA graphics and determine the variable importance for projection (VIP) scores. The number of misclassifications (NMC) was calculated using the R software (v. 3.4.3). Main effects of ozone and herbivory and their interactions were tested by GLM for all individual compounds with a VIP score > 1.0 for component 1.

## Results

### Ozone, herbivory and plant type effects on total volatile emissions and different classes of volatile organic compounds (Fig. 1 and Table 1)

The effects of ozone, herbivory and plant type (wild or cultivated) on total plant volatile emissions were first tested. The results showed that herbivore-feeding increased the total volatile emissions (*P* < 0.001) as well as emissions of several compound groups, including total GLVs (*P* < 0.001), monoterpenes (*P* < 0.001), sesquiterpenes (*P* < 0.001) and N- and S-containing compounds (*P* < 0.05), irrespective of plants being cultivated or wild. Ozone and plant type (wild or cultivated) had no main effect on the volatile emissions. However, an interactive effect was found for ozone and plant type on sesquiterpene emissions (*P* < 0.05), suggesting that wild and cultivated plants did not respond in the same way to elevated ozone.

**Fig. 1:**
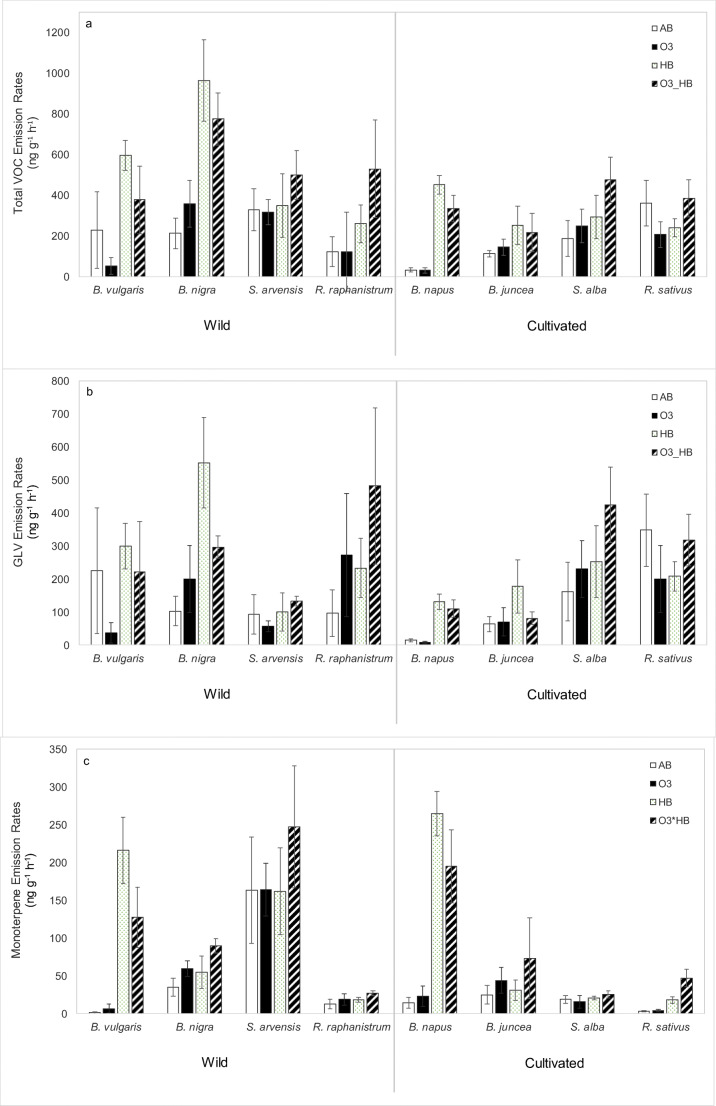

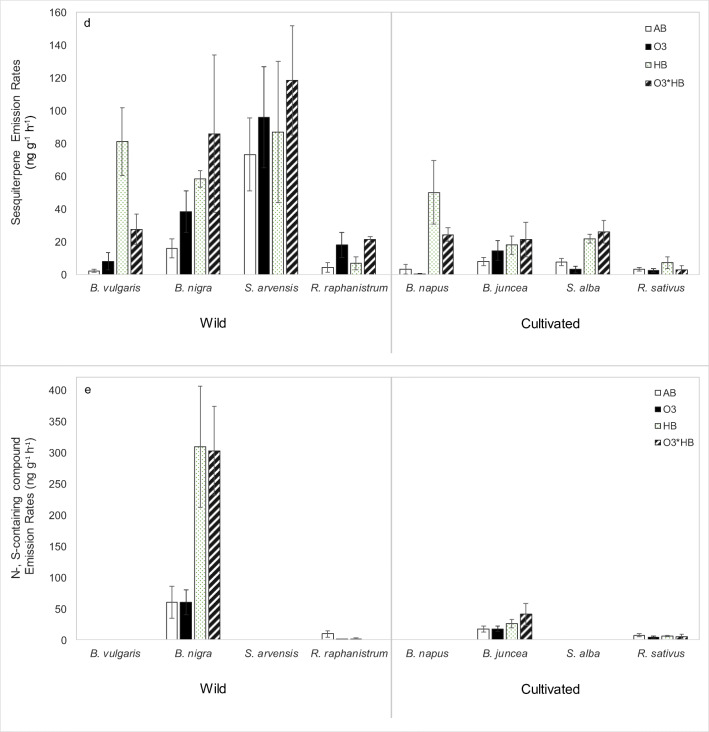
Mean (± SE) emission rates (ng g^−1^ h^−1^) of volatiles emitted by each species under the four treatments. AB, ambient ozone 15 ppb; O3, 80-ppb ozone; HB, ambient ozone and 48 h of herbivore-feeding; O3_HB, 80-ppb ozone and 48 h of herbivore-feeding. **a** Total volatiles. **b** Total green leaf volatiles (GLVs) and methyl salicylate. **c** Total monoterpenes and (*E*)-DMNT. **d** Total sesquiterpene. **e** Total N- and S-containing compounds. Data were statistically analysed using linear mixed model ANOVA for which results are displayed in Table 1

**Table 1:** Results of the linear mixed models ANOVA to test the main effects of herbivory (HB), ozone (O_3_) and plant type (wild or cultivated) (T) and their interactions (HB × O_3_; HB × T; O_3_ × T; HB × O_3_ × T) on volatile emissions. Significant main effects and interactions are italicized. ns = *P* > 0.1

		Herbivory (HB)	Ozone (O_3_)	Type (T)	HB × O_3_	HB × T	O_3_ × T	HB × O_3_ × T
Total VOC	*F*	46.381	0.051	2.725	0.023	1.440	0.007	0.252
	*P* value	< *0.001*	ns	ns	ns	ns	ns	ns
Green leaf volatiles	*F*	40.201	0.571	0.098	0.371	0.045	0.190	0.760
	*P* value	< *0.001*	ns	ns	ns	ns	ns	ns
Monoterpenes	*F*	44.414	1.083	1.448	0.070	0.013	0.217	0.329
	*P* value	< *0.001*	ns	ns	ns	ns	ns	ns
Sesquiterpenes	*F*	41.134	0.220	3.832	0.217	0.570	4.668	0.540
	*P* value	< *0.001*	ns	0.099	ns	ns	*0.033*	ns
N-, and S-containing compounds	*F*	4.477	1.088	0.013	0.050	1.609	0.003	0.861
	*P* value	*0.036*	ns	ns	ns	ns	ns	ns

### Ozone and herbivory effects on the volatile emissions for each species

To further investigate the effects of ozone and herbivore-feeding on volatile emissions, we ran two-way ANOVA on total volatile emissions and compound groups for each species separately (Table 2). Through the use of PLS-DA (Online Resources 3 and 4), we were able to determine the compounds most representative of the variance and test on them the effects of ozone and herbivore-feeding (Table 3).

**Table 2:** Results of the two-way ANOVA to study main effects of herbivory (HB), ozone (O_3_), and their interactive (O_3_ × HB) effect on volatile emissions for each plant species. Significant main effect and interactions are embolden. ns = *P* > 0.1

Type	Species	HB	O_3_	O_3_ × HB
Total VOC				
Wild	*B. vulgaris*	**0.006**	ns	ns
	*B. nigra*	**< 0.001**	ns	ns
	*S. arvensis*	ns	ns	ns
	*R. raphanistrum*	ns	ns	ns
Cultivated	*B. juncea*	ns	ns	ns
	*B. napus*	**< 0.001**	ns	ns
	*S. alba*	0.057	ns	ns
	*R. sativus*	ns	ns	ns
GLVs				
Wild	*B. vulgaris*	**0.005**	ns	ns
	*B. nigra*	**0.002**	ns	ns
	*S. arvensis*	ns	ns	ns
	*R. raphanistrum*	ns	ns	ns
Cultivated	*B. juncea*	0.073	ns	ns
	*B. napus*	**< 0.001**	ns	ns
	*S. alba*	0.065	ns	ns
	*R. sativus*	ns	ns	ns
Monoterpenes				
Wild	*B. vulgaris*	**< 0.001**	ns	ns
	*B. nigra*	ns	**0.047**	ns
	*S. arvensis*	ns	ns	ns
	*R. raphanistrum*	ns	ns	ns
Cultivated	*B. juncea*	ns	ns	ns
	*B. napus*	**< 0.001**	ns	ns
	*S. alba*	ns	ns	ns
	*R. sativus*	**< 0.001**	ns	ns
Sesquiterpenes				
Wild	*B. vulgaris*	**< 0.001**	ns	**0.027**
	*B. nigra*	**0.014**	ns	ns
	*S. arvensis*	ns	ns	ns
	*R. raphanistrum*	ns	**0.025**	ns
Cultivated	*B. juncea*	ns	ns	ns
	*B. napus*	**< 0.001**	ns	ns
	*S. alba*	**< 0.001**	ns	ns
	*R. sativus*	ns	ns	ns
Nitrogen-, and sulphur-containing compounds				
Wild	*B. vulgaris*	-	-	-
	*B. nigra*	**< 0.001**	ns	ns
	*S. arvensis*	-	-	-
	*R. raphanistrum*	ns	0.096	ns
Cultivated	*B. juncea*	ns	ns	ns
	*B. napus*	-	-	-
	*S. alba*	-	-	-
	*R. sativus*	ns	ns	ns

**Table 3:** Mean (± SE) emission rates (ng g^−1^ h^−1^) for the compounds with PLS-DA VIP scores over or equal to 1.0 for component 1. The C1 and C2 in brackets indicate VIP scores for component 1 and component 2, respectively. The main effects of ozone (O_3_), herbivory (HB), and their interaction (O_3_ × HB) were tested with generalised linear models. RI indicates retention indices of unknown compounds, and *n* the number of replicates. Simple main effects and interactions are emboldened. ns = *P* > 0.1

	AB	O_3_	HB	O_3_+HB	Ozone (O_3_)	Herbivory (HB)	HB × O_3_
*B. vulgaris*	*n* = 4	*n* = 4	*n* = 4	*n* = 4			
(*E*)-β-Ocimene (C1:1.6; C2:1.1)	0.0 ± 0.0	1.1 ± 0.7	25.1 ± 5.9	16.5 ± 3.1	ns	**0.022**	ns
(*Z*)-β-Ocimene (C1:1.5; C2:1.0)	0.0 ± 0.0	1.5 ± 1.5	48.9 ± 11.5	38.5 ± 12.1	ns	0.057	ns
(*E*)-DMNT (C1:1.3; C2:0.9)	0.0 ± 0.0	0.0 ± 0.0	140.3 ± 37.3	68.9 ± 35.6	ns	**< 0.001**	ns
α-Bergamotene (C1:1.2; C2:0.9)	0.0 ± 0.0	0.4 ± 0.4	2.2 ± 0.8	1.2 ± 0.3	ns	ns	ns
(*E*,*E*)-α-Farnesene (C1:1.0; C2:0.9)	2.3 ± 0.9	6.9 ± 4.3	61.4 ± 17.0	23.6 ± 7.5	ns	0.084	ns
*B. nigra*	*n* = 5	*n* = 5	*n* = 4	*n* = 5			
(*E*)-DMNT (C1:1.5; C2:1.4)	0.0 ± 0.0	3.0 ± 3.0	26.6 ± 21.9	32.3 ± 6.6	ns	0.090	ns
Sesquiterpene RI 1530 (C1:1.4; C2:1.7)	3.4 ± 1.2	6.4 ± 2.1	23.9 ± 8.9	53.3 ± 45.6	ns	**0.008**	ns
Sesquiterpene RI 1347 (C1:1.4; C2:1.1)	1.1 ± 0.3	1.9 ± 0.5	1.3 ± 0.1	2.2 ± 0.3	ns	ns	ns
Sesquiterpene RI 1409 (C1:1.4; C2:1.1)	1.1 ± 0.3	2.0 ± 0.6	1.8 ± 0.2	2.3 ± 0.3	**0.029**	ns	ns
3-Butenenitrile (C1:1.3; C2:1.4)	0.9 ± 0.4	2.1 ± 1.1	14.5 ± 5.5	13.4 ± 4.4	ns	**0.032**	ns
(*Z*)-γ-Bisabolene (C1:1.3; C2:1.0)	0.2 ± 0.2	0.7 ± 0.2	0.5 ± 0.2	0.9 ± 0.1	ns	ns	ns
1,8-Anhydro-(E)-α-copaene-8-ol (C1:1.3; C2:1.0)	1.5 ± 1.3	5.2 ± 2.5	4.2 ± 1.8	6.7 ± 1.0	ns	ns	ns
(*Z*)-2-Hexenal (C1:1.3; C2:1.0)	3.0 ± 3.0	9.3 ± 6.5	113.2 ± 31.0	38.3 ± 5.4	ns	ns	ns
Caryophyllene (C1:1.3; C2:1.0)	0.5 ± 0.3	3.4 ± 2.3	3.3 ± 1.5	3.8 ± 0.9	ns	ns	ns
Hexanol (C1:1.2; C2:1.0)	0.1 ± 0.1	0.3 ± 0.1	1.2 ± 0.2	0.8 ± 0.2	ns	ns	ns
β-Pinene (C1:1.2; C2:1.0)	0.3 ± 0.1	0.5 ± 0.1	0.3 ± 0.1	0.5 ± 0.1			
α-Longipinene (C1:1.1; C2:1.1)	1.1 ± 0.4	2.7 ± 1.1	1.3 ± 0.5	2.8 ± 0.3	ns	ns	ns
Sesquiterpene RI 1374 (C1:1.1; C2:1.1)	3.0 ± 0.7	5.4 ± 1.6	3.7 ± 0.5	5.6 ± 0.6	**0.013**	ns	ns
Allyl isothiocyanate (C1:1.0; C2:0.9)	58.0 ± 24.9	57.9 ± 19.5	281.3 ± 88.9	285.5 ± 65.9	ns	**0.008**	ns
*S. arvensis*	*n* = 5	*n* = 5	*n* = 3	*n* = 4			
(*E*)-3-Hexenol (C1:1.6; C2:1.7)	4.4 ± 3.6	10.4 ± 3.7	9.0 ± 4.2	17.4 ± 2.3	ns	ns	ns
(*E*)-3-Hexenyl acetate (C1:1.6; C2:1.7)	20.5 ± 15.8	29.2 ± 12.3	49.5 ± 29.9	75.3 ± 26.0	ns	ns	ns
(*Z*)-2-Hexenal (C1:1.5; C2:1.4)	5.6 ± 4.5	7.5 ± 4.1	16.6 ± 9.1	23.2 ± 5.4	ns	ns	ns
Sesquiterpene RI 1411 (C1:1.4; C2:1.3)	7.7 ± 3.4	13.1 ± 5.1	13.3 ± 6.5	16.8 ± 4.2	ns	ns	ns
Caryophyllene (C1:1.4; C2:1.3)	3.0 ± 2.0	14.4 ± 8.5	5.0 ± 2.4	3.8 ± 1.0	ns	ns	ns
1,8-Cineole (C1:1.2; C2:1.1)	8.8 ± 8.8	18.0 ± 11.5	11.2 ± 6.1	42.1 ± 21.9	ns	ns	ns
*R. raphanistrum*	*n* = 3	*n* = 4	*n* = 4	*n* = 4			
Methyl sulfone (C1:1.6; C2:1.7)	9.3 ± 5.2	0.3 ± 0.3	1.5 ± 1.1	0.0 ± 0.0	ns	ns	ns
Sesquiterpene RI 1376 (C1:1.6; C2:1.4)	0.0 ± 0.0	2.2 ± 0.9	0.6 ± 0.4	2.5 ± 0.2	0.086	ns	ns
Sesquiterpene RI 1416 (C1:1.4; C2:1.1)	0.0 ± 0.0	1.2 ± 0.7	1.9 ± 1.4	2.4 ± 0.3	ns	ns	ns
(*E*)-DMNT (C1:1.4; C2:1.2)	0.0 ± 0.0	0.0 ± 0.0	1.2 ± 1.2	6.2 ± 4.1	ns	ns	ns
Caryophyllene (C1:1.3; C2:1.1)	0.0 ± 0.0	0.2 ± 0.2	0.6 ± 0.4	2.0 ± 0.9	ns	ns	ns
(*E*)-3-Hexenol (C1:1.3; C2:1.1)	3.8 ± 3.8	25.6 ± 14.9	23.5 ± 8.7	34.6 ± 15.6	ns	ns	ns
Dehydroaromadendrene (C1:1.3; C2:1.2)	1.0 ± 1.0	5.5 ± 2.3	1.4 ± 0.8	5.4 ± 0.5	ns	ns	ns
β-Pinene (C1:1.2; C2:1.0)	0.0 ± 0.0	0.3 ± 0.2	0.2 ± 0.1	0.4 ± 0.2	ns	ns	ns
Sesquiterpene RI 1386 (C1:1.2; C2:1.0)	0.3 ± 0.3	1.1 ± 0.7	0.3 ± 0.3	1.2 ± 0.1	ns	ns	ns
Δ-3-Carene (C1:1.1; C2:1.7)	0.0 ± 0.0	0.1 ± 0.1	0.0 ± 0.0	0.2 ± 0.2	0.082	ns	ns
*B. juncea*	*n* = 5	*n* = 5	*n* = 5	*n* = 5			
β-Pinene (C1:2.9; C2:1.7)	0.1 ± 0.1	0.4 ± 0.1	0.3 ± 0.1	0.5 ± 0.2	ns	ns	ns
(*Z*)-2-Hexenal (C1:2.8; C2:1.6)	0.0 ± 0.0	8.8 ± 8.8	5.3 ± 5.3	7.7 ± 3.7	ns	ns	ns
Tert-Butyl isothiocyanate (C1:1.4; C2:1.0)	12.5 ± 3.2	13.0 ± 3.6	15.7 ± 4.2	32.1 ± 14.2	ns	**0.026**	ns
(*E*)-Ocimene (C1:1.3; C2:1.0)	0.8 ± 0.6	2.0 ± 0.8	0.6 ± 0.6	3.6 ± 2.6	ns	ns	ns
Benzonitrile (C1:1.3; C2:1.0)	1.5 ± 0.8	0.9 ± 0.3	3.8 ± 1.7	0.5 ± 0.3	ns	ns	ns
Sesquiterpene RI 1436 (C1:1.2; C2:1.5)	0.4 ± 0.3	1.3 ± 0.4	1.5 ± 0.6	0.6 ± 0.4	ns	ns	ns
Allyl isothiocyanate (C1:1.2; C2:0.9)	3.1 ± 1.0	3.6 ± 0.8	6.1 ± 1.7	8.4 ± 3.7	ns	0.072	ns
(*E*)-3-Hexenol (C1:1.1; C2:0.8)	4.3 ± 1.4	5.7 ± 1.6	41.4 ± 28.2	12.5 ± 3.5	0.062	**0.003**	**0.039**
Δ-3-Carene (C1:1.1; C2:1.0)	2.3 ± 1.1	4.6 ± 1.8	3.4 ± 1.4	8.1 ± 6.0	ns	ns	ns
*B. napus*	*n* = 5	*n* = 5	*n* = 5	*n* = 5			
β-Myrcene (C1:1.7; C2:1.2)	0.0 ± 0.0	0.2 ± 0.2	6.4 ± 1.3	6.7 ± 1.0	ns	**0.006**	ns
(*Z*)-2-Hexenal (C1:1.5; C2:1.6)	0.0 ± 0.0	0.0 ± 0.0	1.2 ± 1.2	5.8 ± 2.4	ns	ns	ns
1,8-Cineole (C1:1.4; C2:1.0)	0.4 ± 0.2	0.6 ± 0.2	6.7 ± 1.3	10.5 ± 4.6	ns	0.051	ns
α-Selinene (C1:1.3; C2:0.9)	0.0 ± 0.0	0.0 ± 0.0	9.1 ± 3.7	5.6 ± 0.5	ns	**< 0.001**	ns
(*E*)-DMNT (C1:1.3; C2:0.9)	0.0 ± 0.0	0.0 ± 0.0	176.9 ± 14.0	117.6 ± 36.2	ns	**< 0.001**	ns
Sesquiterpene RI 1438 (C1:1.3; C2:1.0)	0.1 ± 0.1	0.2 ± 0.2	2.7 ± 0.3	1.4 ± 0.6	ns	**< 0.001**	ns
(*E*,*E*)-α-Farnesene (C1:1.3; C2:0.9)	0.0 ± 0.0	0.0 ± 0.0	9.2 ± 2.4	4.6 ± 0.8	ns	**<0.001**	ns
α-Thujene (C1:1.2; C2:0.8)	0.2 ± 0.2	0.3 ± 0.2	4.8 ± 0.9	5.6 ± 0.7	ns	**0.050**	ns
Menthol (C1:1.1; C2:1.2)	4.4 ± 2.9	2.1 ± 1.3	5.3 ± 5.3	0.0 ± 0.0	ns	ns	ns
β-Pinene (C1:1.1; C2:0.9)	0.0 ± 0.0	0.0 ± 0.0	1.1 ± 0.2	1.0 ± 0.2	ns	**0.030**	ns
Sesquiterpene RI 1506 (C1:1.1; C2:1.0)	0.0 ± 0.0	0.0 ± 0.0	2.9 ± 0.8	1.7 ± 0.3	ns	**< 0.001**	ns
(*E*)-3-Hexenol (C1:1.0; C2:1.0)	0.0 ± 0.0	0.0 ± 0.0	11.5 ± 2.4	9.5 ± 2.6	ns	**< 0.001**	ns
*S. alba*	*n* = 5	*n* = 4	*n* = 4	*n* = 5	*n* = 5		
Sabinene (C1:1.6; C2:1.4)	0.0 ± 0.0	0.0 ± 0.0	1.3 ± 0.5	3.4 ± 0.5	ns	**< 0.001**	ns
α-Pinene (C1:1.6; C2:1.7)	0.0 ± 0.0	1.2 ± 0.7	0.5 ± 0.5	1.1 ± 0.3	ns	ns	ns
(*E*)-DMNT (C1:1.5; C2:1.2)	0.0 ± 0.0	0.0 ± 0.0	0.9 ± 0.9	2.0 ± 0.8	ns	0.055	ns
1,8-Cineole (C1:1.4; C2:1.2)	0.1 ± 0.1	5.2 ± 5.2	0.7 ± 0.3	2.2 ± 0.4	ns	ns	ns
Germacrene-D (C1:1.4; C2:1.3)	0.0 ± 0.0	0.0 ± 0.0	12.4 ± 2.9	16.4 ± 5.5	ns	**< 0.001**	ns
Hexanal (C1:1.1; C2:1.0)	0.1 ± 0.1	1.1 ± 0.7	1.2 ± 0.7	17.1 ± 6.0	ns	ns	ns
Methyl salicylate (C1:1.0; C2:0.9)	0.0 ± 0.0	0.0 ± 0.0	1.1 ± 0.9	1.8 ± 0.8	ns	**0.050**	ns
*R. sativus*	*n* = 5	*n* = 4	*n* = 5	*n* = 4			
(*Z*)-β-ocimene (C1:2.0; C2:1.9)	2.9 ± 0.6	0.4 ± 0.4	6.3 ± 0.9	5.2 ± 1.2	ns	ns	ns
β-Pinene (C1:1.7; C2:1.5)	0.0 ± 0.0	1.9 ± 0.9	2.6 ± 2.6	2.3 ± 1.0	ns	ns	ns
(*E*)-DMNT (C1:1.5; C2:1.3)	0.0 ± .0.0	0.0 ± 0.0	8.3 ± 3.1	36.5 ± 9.5	ns	ns	ns
Methyl isothiocyanate (C1:1.2; C2:1.1)	7.0 ± 2.3	4.0 ± 2.2	5.7 ± 0.8	4.9 ± 3.3	ns	ns	ns
Limonene (C1:1.2; C2:1.2)	0.2 ± 0.2	0.7 ± 0.7	0.0 ± 0.0	1.1 ± 0.5	ns	ns	ns
Δ-3-Carene (C1:1.1; C2:1.0)	0.0 ± 0.0	0.0 ± 0.0	0.2 ± 0.1	0.6 ± 0.3	ns	0.099	ns

#### Ozone and herbivore effects on the volatile emissions of wild plants

*B. vulgaris* increased total volatile, GLV and mono- and sesquiterpene emissions in response to herbivory (Table 2), especially (*E*)-β-ocimene (*P* < 0.05), (*Z*)-β-ocimene (0.05 < *P* < 0.1) and (*E*)-DMNT (*P* < 0.001) emissions (Table 3). Results also highlighted an interactive effect between ozone and herbivory on sesquiterpene emissions of *B. vulgaris* (*P* < 0.05), in which the quantity of sesquiterpenes emitted in response to the ozone and herbivory treatment was 63% lower than that in the herbivore treatment with ambient ozone (Fig. 1d).

*B. nigra* emitted the most N- and S-containing compounds (Fig. 1e), with eight different compounds detected as being emitted (Online Resource 2). Herbivore-feeding significantly increased emissions of total N- and S-containing compounds (*P* < 0.001), which included significant induction of 3-butenenitrile (*P* < 0.05) and allyl isothiocyanate (*P* < 0.01). *B. nigra* also had herbivore-induced increases in GLV and sesquiterpene emissions, including emissions of the unknown sesquiterpene with RI 1530 (*P* < 0.01). *B. nigra* increased monoterpene emissions by 51% (*P* < 0.05, Fig. 1c) under elevated ozone and increased two unknown sesquiterpenes with RI 1530 (*P* < 0.05) and RI 1374 (*P* < 0.05, Table 3).

Analyses for *S. arvensis* did not show significant differences.

There was no herbivory effect for *R. raphanistrum*. Under elevated ozone, *R. raphanistrum* had three times higher sesquiterpene emissions (*P* < 0.05, Fig. 1d). *R. raphanistrum* tended to have increased Δ-3-carene (0.05 < *P* < 0.1) and unknown sesquiterpene RI 1376 emissions (0.05 < *P* < 0.1, Table 3) under elevated ozone.

#### Ozone and herbivore effects on the volatile emissions of cultivated plants

In *B. juncea*, herbivory increased emission rates of tert-butyl isothiocyanate (*P* < 0.05) and (*E*)-3-hexenol (*P* < 0.01) but elevated ozone reduced emission of (*E*)-3-hexenol to herbivory as shown by the interactive effect found between ozone and herbivory (Table 3)*.*

*B. napus* increased total VOC emissions, GLVs, monoterpenes, sesquiterpenes and N- and S-containing compounds in response to herbivore-feeding (Table 2). *B. napus* emitted the most diverse range of herbivore-induced volatiles. Herbivory increased β-myrcene (*P* < 0.01), α-selinene (*P* < 0.001), (*E*)-DMNT (*P* < 0.001), two unknown sesquiterpenes RI 1438 (*P* < 0.001) and RI 1506.5 (*P* < 0.001), (*E*,*E*)-α-farnesene (*P* < 0.001), α-thujene (*P* < 0.05), β-pinene (*P* < 0.05), (*E*)-3-hexenol (*P* < 0.001) and 1,8-cineole (0.05 < *P* < 0.1) (Table 3). There was no ozone effect for this species.

*S. alba* increased sesquiterpene emissions (*P* < 0.001) in response to herbivory (Table 2), especially the germacrene-D (*P* < 0.001). *S. alba* also increased sabinene (P<0.001) and  MeSA (*P* < 0.05) emissions in response to herbivory (Table 3). *R. sativus* increased monoterpene emissions (*P* < 0.001) in response to herbivory (Table 2). There was no ozone effect for both species.

## Discussion

### Effects of herbivore-feeding on volatile emissions

In accordance with our hypothesis, herbivore-feeding increased the volatile emissions in most of the *Brassicaceae* species studied. The responses were characterised mainly by increases in terpene and GLV emissions, and as a consequence, the total volatile emissions. Volatiles such as terpenes and GLVs are secondary metabolites that are emitted constitutively in relatively small quantities and induced by mechanical damage and herbivore-feeding (Dudareva 2013; Arimura et al. 2000). Their emission by plants is the result of the activation of different biosynthesis pathways. GLVs are produced via the lipoxygenase pathway and are synthesized by plants in response to mechanical damage or herbivore-feeding (Matsui 2006), which is consistent with the herbivore-induced increases in those compounds observed in our study. Terpenoids are also induced by herbivore-feeding, and they constitute the largest and most diverse class of secondary metabolites and are synthesized via the methylerythritol phosphate pathway and the mevalonate pathway (Dudareva 2013). This is also consistent with the herbivore-induced increase in terpenoids observed in this study.

Terpenes and GLVs are both involved in direct defence against herbivores through effects on their growth and development or in indirect defence through the recruitment of natural enemies of herbivores (McCormick et al. 2012; Arimura et al. 2009). Glucosinolates, characteristic to the *Brassicaceae*, are degraded upon tissue damage into volatile N- and S-containing compounds (Hopkins et al. 2009), which can also play major roles in plant defences (Agrawal and Kurashige 2003). In this study, *B. nigra* had the greatest emission of HIPVs and the greatest diversity and quantity of N- and S-containing compounds in their HIPV blend. Another wild species, *R. raphanistrum*, and two cultivated species, *B. juncea* and *R. sativus*, constitutively emitted N- and S-containing compounds. *Brassica juncea*, which is the result of hybridisation between wild *B. rapa* and *B. nigra*, emitted lower levels of N- and S-containing compounds than *B. nigra*. To produce cultivars with high yield and palatability, crop breeders have selected for decreased content of glucosinolates (Mithen et al. 1987), resulting in lower amounts of isothiocyanates emitted in response to herbivory compared to their wild relatives (Gols et al. 2011). The results presented here also showed that *B. napus* emitted the lowest amount of constitutive volatiles but had the greatest diversity of HIPVs. This result is in agreement with a recent study comparing plant defences in wild and cultivated tomatoes (Paudel et al. 2019). They found that all tomato species (cultivated and wild) responded to herbivore-feeding by increasing their level of volatile emissions. However, such as for *B. napus*, they found that cultivated tomatoes *Solanum lycopersicum* had the overall strongest response to herbivory by increasing HIPVs more than the wild tomato *S. pimpinellifolium.*

Interestingly, *S. arvensis* and *R. raphanistrum* did not show any changes in volatile emissions in response to herbivore-feeding. This was an unexpected result and verified in follow-up tests (data not included). Plants can adapt their responses to herbivory according to the stress intensity and the stress duration. For instance, plants can induce volatiles differently if they are attacked by young larvae (first and second instar) and more mature caterpillars (Dicke 1999). Moreover, corn damaged by first to third instar larvae of *Pseudaletia separata* emitted HIPVs, but emissions were drastically reduced when plants were damaged with later instars (Takabayashi et al. 1995). It is possible that the stress applied in this study was not intense enough to induce a response in *S. arvensis* and *R. raphanistrum*, possibly due to those plants being the best defended, but those hypotheses need further investigation.

### Effects of ozone on volatile emissions

Ozone is a phytotoxic tropospheric pollutant that triggers the formation of reactive oxygen species (ROS) in plant cells (Vainonen and Kangasjärvi 2015). Emission of volatile compounds by plants, especially terpenes, has been shown to have antioxidant function and provide some protection against oxidative damage in plant cells (Vickers et al. 2009). For instance, exposure of plants to elevated ozone led to an increase of monoterpenes in *Nicotiana tabacum* (Heiden et al. 1999), *Pinus sylvestris* and *Populus nigra* (Fares et al. 2008). In our study, elevated ozone had a smaller and less consistent effect on volatile emissions than herbivore-feeding. It was notable that the significant effects of ozone on volatile emissions were all found in the wild plants, specifically *B. nigra* and *R. raphanistrum*. Elevated ozone increased emissions of total monoterpenes and two unknown sesquiterpenes (RI 1347 and RI 1409) of *B. nigra* and the total sesquiterpene emissions of *R. raphanistrum*.

In the treatment with both ozone and herbivore-feeding, there was a significant interactive effect between the two stresses on the sesquiterpene emissions of *B. vulgaris*. In this case, the emissions were lower than those induced by herbivore-feeding alone. Effects of ozone exposure on the volatiles emitted in response to herbivory have been observed several times. An increase in HIPVs under elevated ozone has been reported in *Nicotiana tabacum* (Beauchamp et al. 2005) and *B. nigra* (Khaling et al. 2016; Papazian et al. 2016). Khaling et al. (2016) observed higher *P. brassicae*–induced volatile emissions in *B. nigra* under 120-ppb ozone, but not under 70-ppb ozone, suggesting that there could be a threshold to reach to observe an enhancement of the herbivore response. It is notable that Khaling et al. (2016) actually observed slightly lower levels of HIPV emissions at 70 ppb than at ambient ozone levels of approximately 15 ppb, suggesting that in this study, in which 80 ppb was used, a critical threshold for strong ozone effects may not have been reached. A decrease in HIPV emissions under 100-ppb ozone has also been reported in *B. napus* with a 45% reduction in terpenoid emissions (Himanen et al. 2009). As mentioned above, plants respond to herbivore-feeding by activating signalling pathways to produce volatiles. The ROS in plant cells induced by ozone exposure can activate the salicylic acid pathway (Caarls et al. 2015; Mur et al. 2008) and cause defensive responses in plants similar to those occurring in response to pathogen infections (Pinto et al. 2007; Kangasjarvi et al. 2005). Thus, ozone could influence the complex regulatory signalling pathways involved in plant responses to herbivory, leading to these changes in volatiles and especially HIPVs (Bidart-Bouzat and Imeh-Nathaniel 2008). In this study, the ozone level used was representative of realistic high current ozone levels although higher peak levels can be seen, usually in summer time in high ozone regions (Mills et al. 2018). It is clear from this study that there is variation in how closely related *Brassicaceous* plants respond to elevated ozone levels and indeed the extent of their responses to herbivore-feeding. The present study did not show any interactive effects of ozone and herbivory in cultivated plants. It is possible that the processes of selection and breeding could have affected the ways in which plants respond to abiotic stress. Cultivated plants could also be more tolerant to elevated ozone than wild plants, explaining the lack of ozone effects on their volatile emissions.

## Conclusions

The results indicated that most of the wild and cultivated plants increased volatile emissions in response to herbivore-feeding. The extent of this response differed between species and was absent in *S. arvensis* and *R. raphanistrum*. Ozone increased or decreased the monoterpene and sesquiterpene emissions in three of the four wild plants studied and there was an interactive effect of ozone and herbivory on the sesquiterpene emissions of *B. vulgaris*. The results revealed that wild plants might in general be more sensitive to elevated ozone than the cultivated plants, although the mechanisms underlying this observation need to be tested. A universal prediction of how elevated ozone will affect plant volatile profiles and defences is not possible due to the variation in responses. However, it would seem feasible that wild plants could be the most affected by elevated ozone and attention should be made to ensuring that wild plants are not neglected in future studies.

## Electronic supplementary material

ESM 1 (DOCX 585 kb)
