# Supplementary material for: Does ozone exposure affect herbivore-induced plant volatile emissions differently in wild and cultivated plants?
Source: Environ Sci Pollut Res Int. 2020 May 28;27(24):30448–59. doi: 10.1007/s11356-020-09320-z (PMC7378123; doi:10.1007/s11356-020-09320-z)
Supplement: Supplementary file 1 — (DOCX 585 kb) [file 11356_2020_9320_MOESM1_ESM.docx]

**Does ozone exposure affect herbivore induced plant volatile emissions differently in wild and cultivated plants?**

Agnès Brosset^*^, Amélie Saunier, Minna Kivimäenpää and James D. Blande

Department of Environmental and Biological Sciences, University of Eastern Finland, Kuopio FIN-70211, Finland

*Corresponding author

[agnes.brosset@uef.fi](mailto:agnes.brosset@uef.fi)

**Online resource 1**: The experimental design consisted of plants exposed for seven days to ambient (15-20ppb) or elevated (80ppb) ozone (O_3_). After five days, half of the plants under each exposure regime were infested with 20 third instar *Plutella xylostella* larvae for 48h (HB) and (O_3_+HB). Ozone was elevated to 80ppb at 7:00 and reduced to 30ppb at 20:00, to follow a realistic daily ozone oscillation for elevated ozone treatments (O_3_ and O_3_+HB). The ozone ranged between 15 and 20ppb in the ambient ozone treatments (AB and HB).

**Online resource 2.** Volatile emission rates (mean ± S.E.) (ng.g^-1^.h^-1^) of eight species of *Brassicaceae* exposed to ambient ozone 15ppb (AB); 80ppb ozone (O_3_); ambient ozone and 48h of herbivore-feeding (HB)_;_ 80ppb ozone and 48h of herbivore-feeding (O_3_+HB). Exposure to ambient or elevated ozone was for five days prior to *Plutella xylostella* larvae being added as the herbivore-feeding treatment. RI indicates retention indices, which were calculated for unknown compounds based on a standard series of alkanes C8-C20.

| Compounds | **AB** | **O_3_** | **HB** | **O_3_+HB** |
| --- | --- | --- | --- | --- |
| ***Barbarea vulgaris*** | **n=4** | **n=4** | **n=4** | **n=4** |
| **Monoterpenes** |  |  |  |  |
| Limonene | 1.2±1.2 | 3.7±3.7 | 2.1±2.1 | 3.2±1.8 |
| (*E*)-β-Ocimene | 0.0±0.0 | 1.1±0.7 | 25.1±5.9 | 16.5±3.1 |
| (*Z*)-β-Ocimene | 0.0±0.0 | 1.5±1.5 | 48.9±11.5 | 38.5±12.1 |
| **Homoterpene** |  |  |  |  |
| (*E*)-DMNT | 0.0±0.0 | 0.0±0.0 | 140.3±37.3 | 68.9±35.6 |
| **Sesquiterpenes** |  |  |  |  |
| Caryophyllene | 0.0±0.0 | 0.5±0.5 | 16.8±3.3 | 2.2±1.7 |
| α-Bergamotene | 0.0±0.0 | 0.4±0.4 | 2.2±0.8 | 1.2±0.3 |
| (*E,E*)-α-Farnesene | 2.3±0.9 | 6.9±4.3 | 61.4±17.0 | 23.6±7.5 |
| Farnesol | 0.0±0.0 | 0.3±0.2 | 0.9±0.3 | 0.6±0.3 |
| **GLVs** |  |  |  |  |
| (*E*)-3-Hexenal | 58.4±58.4 | 0.0±0.0 | 21.3±12.9 | 160.3±147 |
| (*E*)-3-Hexenol | 80.3±70.4 | 13.9±12.1 | 40.0±10.1 | 17.0±4.3 |
| (*E*)-3-Hexenyl acetate | 86.3±61.1 | 23.3±18.9 | 238.3±57.8 | 44.9±8.8 |
| ***Brassica nigra*** | **n=5** | **n=5** | **n=4** | **n=5** |
| **Monoterpenes** |  |  |  |  |
| α-Pinene | 1.5±0.4 | 2.5±0.2 | 0.9±0.4 | 2.2±0.5 |
| β-Pinene | 0.3±0.1 | 0.5±0.1 | 0.3±0.1 | 0.5±0.1 |
| Δ-3-Carene | 1.6±0.7 | 1.8±0.3 | 0.9±0.5 | 1.9±0.6 |
| Limonene | 28.5±10.1 | 48.5±8 | 23.9±5.2 | 48.7±13.7 |
| (*E*)-Ocimene | 1.1±0.6 | 1.2±0.6 | 0.9±0.4 | 2.0±0.6 |
| Menthol | 1.2±0.2 | 1.5±0.2 | 1.2±0.1 | 1.5±0.2 |
| **Homoterpene** |  |  |  |  |
| (*E*)-DMNT | 0.0±0.0 | 3.0±3.0 | 26.6±21.9 | 32.3±6.6 |
| **Sesquiterpenes** |  |  |  |  |
| Sesquiterpene RI 1347 | 1.1±0.3 | 1.9±0.5 | 1.3±0.1 | 2.2±0.3 |
| Sesquiterpene RI 1364 | 0.8±0.4 | 3.6±1.3 | 2.3±0.8 | 0.3±0.3 |
| Sesquiterpene RI 1374 | 3.0±0.7 | 5.4±1.6 | 3.7±0.5 | 5.6±0.6 |
| α-Longipinene | 1.1±0.4 | 2.7±1.1 | 1.3±0.5 | 2.8±0.3 |
| Sesquiterpene RI 1409 | 1.1±0.3 | 2.0±0.6 | 1.8±0.2 | 2.3±0.3 |
| Caryophyllene | 0.5±0.3 | 3.4±2.3 | 3.3±1.5 | 3.8±0.9 |
| Sesquiterpene RI 1436 | 0.1±0.1 | 0.0±0.0 | 1.7±0.2 | 0.2±0.1 |
| 1,8-Anhydro-(*E*)-α-copaene-8-ol | 1.5±1.3 | 5.2±2.5 | 4.2±1.8 | 6.7±1.0 |
| Sesquiterpene RI 1530 | 3.4±1.2 | 6.4±2.1 | 23.9±8.9 | 53.3±45.6 |
| (*Z*)-γ-Bisabolene | 0.2±0.2 | 0.7±0.2 | 0.5±0.2 | 0.9±0.1 |
| **GLVs** |  |  |  |  |
| (*E*)-3-Hexenal | 23.5±23.5 | 57.8±57.8 | 210.7±72.9 | 92.4±24.0 |
| Hexanal | 59.7±13.4 | 75.7±17.2 | 162.4±31.9 | 139.8±17.6 |
| (*Z*)-2-Hexenal | 3.0±3.0 | 9.3±6.5 | 113.2±31.0 | 38.3±5.4 |
| (*E*)-3-Hexenol | 8.0±5.8 | 19.0±14.2 | 25.1±5.2 | 12.2±3.8 |
| Hexanol | 0.1±0.1 | 0.3±0.1 | 1.2±0.2 | 0.8±0.2 |
| (*E*)-3-Hexenyl acetate | 8.1±5.1 | 38.0±32.6 | 39.4±18.0 | 12.8±7.3 |
| Hexyl acetate | 0.0±0.0 | 0.0±0.0 | 0.9±0.6 | 0.3±0.3 |
| **N-/or S-containing compounds** |  |  |  |  |
| Methyl sulphide | 0.4±0.2 | 0.0±0.0 |  | 1.6±0.7 |
| 3-Butenenitrile | 0.9±0.4 | 2.1±1.1 | 14.5±5.4 | 13.4±4.4 |
| Triethylamine | 0.8±0.6 | 1.3±0.5 | 1.1±0.8 | 2.8±1.1 |
| Methyl isothiocyanate | 0.3±0.2 | 0.2±0.1 | 1.1±0.2 | 1.3±0.2 |
| Dimethyl disulphide | 0.3±0.1 | 0.0±0.0 | 0.7±0.2 | 0.9±0.3 |
| Allyl isothiocyanate | 58.0±24.9 | 57.9±19.5 | 281.3±88.9 | 285.5±65.9 |
| Benzonitrile | 0.0±0.0 | 0.2±0.2 | 0.2±0.2 | 0.2±0.2 |
| Benzyl isothiocyanate | 0.0±0.0 | 0.0±0.0 | 10.2±9.9 | 0.0±0.0 |
| ***Brassica juncea*** | **n=5** | **n=5** | **n=5** | **n=5** |
| **Monoterpenes** |  |  |  |  |
| α-Pinene | 1.0±0.4 | 1.9±0.5 | 1.3±0.4 | 3.2±2.2 |
| β-Pinene | 0.1±0.1 | 0.3±0.1 | 0.3±0.1 | 0.5±0.2 |
| Δ-3-Carene | 2.3±1.1 | 4.6±1.8 | 3.4±1.4 | 8.1±6.0 |
| Limonene | 19.2±10.2 | 32.8±13.5 | 23.7±10.7 | 55.3±40.8 |
| (*E*)-Ocimene | 0.8±0.6 | 2.0±0.8 | 0.6±0.6 | 3.6±2.6 |
| Menthol | 1.0±0.4 | 1.9±0.9 | 1.5±0.6 | 2.2±1.5 |
| **Sesquiterpenes** |  |  |  |  |
| Sesquiterpene RI 1435 | 2.4±0.8 | 3.5±1.5 | 3.1±1.3 | 4.5±2.6 |
| α-Longipinene | 0.8±0.4 | 2.0±0.9 | 1.7±0.7 | 2.5±1.7 |
| Sesquiterpene RI 1402 | 0.1±0.1 | 0.3±0.3 | 0.9±0.4 | 0.0±0.0 |
| Sesquiterpene RI 1409 | 0.6±0.3 | 1.9±0.8 | 1.9±0.8 | 3.3±2.4 |
| Sesquiterpene RI 1428 | 0.1±0.1 | 0.0±0.0 | 1.0±0.3 | 0.3±0.2 |
| Longifolene | 0.5±0.2 | 0.4±0.2 | 1.1±0.3 | 0.8±0.2 |
| Sesquiterpene RI 1436 | 0.4±0.3 | 1.3±0.4 | 1.5±0.6 | 0.6±0.4 |
| Caryophyllene | 1.0±0.2 | 1.3±0.6 | 3.7±0.6 | 4.7±1.6 |
| **GLVs** |  |  |  |  |
| Hexanal | 38.9±24.3 | 44.4±44.4 | 22.5±22.5 | 14.5±14.5 |
| (*Z*)-2-Hexenal | 0.0±0.0 | 8.8±8.8 | 5.3±5.3 | 7.7±3.7 |
| (*E*)-3-Hexenol | 4.3±1.4 | 5.7±1.6 | 41.4±28.2 | 12.5±3.5 |
| (*E*)-3-Hexenyl acetate | 19.9±9.4 | 11.1±4.7 | 108.2±54.1 | 46.2±10.7 |
| **N-/or S-containing compounds** |  |  |  |  |
| Tert-Butyl isothiocyanate | 12.5±3.2 | 13.0±3.6 | 15.7±4.2 | 32.1±14.2 |
| Allyl isothiocyanate | 3.1±1.0 | 3.6±0.8 | 6.1±1.7 | 8.4±3.7 |
| Benzonitrile | 1.5±0.8 | 0.9±0.3 | 3.8±1.7 | 0.5±0.3 |
| ***Brassica napus oleifera*** | **n=5** | **n=5** | **n=5** | **n=5** |
| **Monoterpenes** |  |  |  |  |
| α-Thujene | 0.2±0.2 | 0.3±0.2 | 4.8±0.9 | 5.6±0.7 |
| α-Pinene | 0.5±0.2 | 1.3±0.8 | 3.4±1.0 | 2.4±0.4 |
| Sabinene | 2.6±1.2 | 1.0±1.0 | 26.3±4.3 | 30.5±4.7 |
| β-Pinene | 0.0±0.0 | 0.0±0.0 | 1.1±0.2 | 1.0±0.2 |
| β-Myrcene | 0.0±0.0 | 0.2±0.2 | 6.4±1.3 | 6.7±1.0 |
| Δ-3-Carene | 0.0±0.0 | 0.6±0.5 | 0.8±1.0 | 0.0±0.0 |
| Limonene | 5.4±3.3 | 16.2±11.5 | 37.5±13.7 | 26.0±5.1 |
| 1,8-Cineole | 0.4±0.2 | 0.6±0.2 | 6.7±1.3 | 10.5±4.6 |
| Menthone | 0.6±0.4 | 0.5±0.3 | 0.9±0.9 | 0.0±0.0 |
| Menthol | 4.4±2.9 | 2.1±1.3 | 5.3±5.3 | 0.0±0.0 |
| **Homoterpene** |  |  |  |  |
| (*E*)-DMNT | 0.0±0.0 | 0.0±0.0 | 176.9±14.0 | 117.6±36.2 |
| **Sesquiterpenes** |  |  |  |  |
| β-Elemene | 0.6±0.6 | 0.0±0.0 | 10.1±2.9 | 6.0±0.7 |
| Sesquiterpene RI 1432 | 0.0±0.0 | 0.2±0.2 | 1.6±1.0 | 0.9±0.4 |
| Sesquiterpene RI 1438 | 0.1±0.1 | 0.2±0.2 | 2.7±0.3 | 1.4±0.6 |
| Caryophyllene | 2.6±2.4 | 0.0±0.0 | 14.8±9.7 | 4.2±4.1 |
| Sesquiterpene RI 1506 | 0.0±0.0 | 0.0±0.0 | 2.9±0.8 | 1.7±0.3 |
| (*E,E*)-α-Farnesene | 0.0±0.0 | 0.0±0.0 | 9.2±2.4 | 4.6±0.8 |
| α-Selinene | 0.0±0.0 | 0.0±0.0 | 9.1±3.7 | 5.6±0.5 |
| **GLVs + MeSA** |  |  |  |  |
| (*Z*)-2-Hexenal | 0.0±0.0 | 0.0±0.0 | 1.2±1.2 | 5.8±2.4 |
| (*E*)-3-Hexenol | 0.0±0.0 | 0.0±0.0 | 11.5±2.4 | 9.5±2.6 |
| (*E*)-3-Hexenyl acetate | 14.3±5 | 7.5±3.6 | 99.9±23.1 | 92.3±23.8 |
| Hexyl acetate | 0.0±0.0 | 0.0±0.0 | 3.5±1.1 | 1.7±0.8 |
| Methyl salicylate (MeSA**)** | 0.0±0.0 | 0.0±0.0 | 0.8±0.3 | 0.6±0.4 |
| ***Sinapis arvensis*** | **n=5** | **n=5** | **n=3** | **n=4** |
| **Monoterpenes** |  |  |  |  |
| Monoterpene RI 962 | 0.6±0.4 | 0.8±0.5 | 2.1±0.8 | 3.8±1.4 |
| α-Pinene | 7.3±2.5 | 8.0±2.2 | 7.3±1.8 | 10.5±3.1 |
| β-Pinene | 1.7±0.9 | 2.3±0.9 | 1.4±0.3 | 1.6±0.6 |
| Δ-3-Carene | 10.5±2.7 | 14.3±4.3 | 14.3±5.3 | 20.5±6.5 |
| Monoterpene RI 1031 | 42.2±40.0 | 4.6±1.7 | 6.1±3.5 | 13.5±4.8 |
| Limonene | 92.0±23.1 | 116.4±22.6 | 119.7±46.6 | 155.4±48.6 |
| 1,8-Cineole | 8.8±8.8 | 18.0±11.5 | 11.2±6.1 | 42.1±21.9 |
| **Sesquiterpenes** |  |  |  |  |
| Sesquiterpene RI 1373 | 9.7±2.9 | 15.8±5.1 | 12.0±6.0 | 14.9±5.2 |
| Sesquiterpene RI 1411 | 7.7±3.4 | 13.1±5.1 | 13.3±6.5 | 16.8±4.2 |
| Longipinene | 10.0±3.2 | 11.4±5.6 | 12.6±6.8 | 17.3±5.0 |
| 1,8-Anhydro-(E)-α-copaene-8-ol | 21.4±6.6 | 20.8±9.4 | 22.0±10.8 | 33.0±9.3 |
| Caryophyllene | 3.0±2.0 | 14.4±8.5 | 5.0±2.4 | 3.8±1.0 |
| **GLVs** |  |  |  |  |
| Hexanal | 62.2±58.4 | 9.1±6.5 | 24.3±17.1 | 17.1±6.0 |
| (*Z*)-2-Hexenal | 5.6±4.5 | 7.5±4.1 | 16.6±9.1 | 23.2±5.4 |
| (*E*)-3-Hexenol | 4.4±3.7 | 10.4±3.7 | 9.0±4.2 | 17.4±2.3 |
| (*E*)-3-Hexenyl acetate | 20.5±15.8 | 29.2±12.3 | 49.5±29.9 | 75.3±26.0 |
| ***Sinapis alba*** | **n=5** | **n=4** | **n=4** | **n=5** |
| **Monoterpenes** |  |  |  |  |
| α-Pinene | 0.0±0.0 | 1.2±0.7 | 0.5±0.5 | 1.8±0.8 |
| Sabinene | 0.0±0.0 | 0.0±0.0 | 1.3±0.5 | 3.4±0.5 |
| β-Pinene | 0.3±0.1 | 0.5±0.4 | 0.6±0.3 | 1.1±0.3 |
| Limonene | 18.0±4.8 | 8.3±8.3 | 16.7±1.7 | 14.6±3.4 |
| 1,8-Cineole | 0.1±0.1 | 5.2±5.2 | 0.7±0.3 | 2.2±0.4 |
| **Homoterpene** |  |  |  |  |
| (*E*)-DMNT | 0.0±0.0 | 0.0±0.0 | 0.9±0.9 | 2.0±0.8 |
| **Sesquiterpenes** |  |  |  |  |
| α-Gurjunene | 2.6±0.8 | 0.5±0.5 | 1.4±0.6 | 3.2±0.8 |
| Caryophyllene | 0.4±0.2 | 0.0±0.0 | 3.4±1.2 | 1.9±0.5 |
| Sesquiterpene RI 1448 | 4.5±1.3 | 2.7±1.9 | 4.7±1.4 | 4.8±1.4 |
| Germacrene D | 0.0±0.0 | 0.0±0.0 | 12.4±2.9 | 16.4±5.5 |
| **GLVs + MeSA** |  |  |  |  |
| Hexanal | 0.1±0.1 | 1.1±0.7 | 1.2±0.7 | 1.0±0.5 |
| (*Z*)-2-Hexenal | 0.6±0.6 | 3.8±3.8 | 5.2±4.5 | 5.1±2.9 |
| (*E*)-3-Hexenol | 8.8±3.8 | 23.1±11.6 | 17.3±5.4 | 23.8±4.6 |
| (*E*)-3-Hexenyl acetate | 152.4±84.8 | 201.7±77.3 | 227.3±105.1 | 392.8±110.7 |
| Hexyl acetate | 8.9±8.9 | 7.2±7.2 | 0.0±0.0 | 0.0±0.0 |
| Methyl salicylate | 0.0±0.0 | 0.0±0.0 | 1.1±0.9 | 1.8±0.8 |
| ***Raphanus raphanistrum*** | **n=3** | **n=4** | **n=4** | **n=4** |
| **Monoterpenes** |  |  |  |  |
| α-Pinene | 0.4±0.4 | 0.7±0.4 | 0.0±0.0 | 0.8±0.4 |
| β-Pinene | 0.0±0.0 | 0.3±0.2 | 0.2±0.1 | 0.4±0.2 |
| Δ-3-Carene | 0.0±0.0 | 0.1±0.1 | 0.0±0.0 | 0.2±0.2 |
| Limonene | 7.7±4.0 | 13.6±8.0 | 8.4±3.0 | 11.3±5.5 |
| (*Z*)-β-Ocimene | 1.3±1.1 | 0.4±0.4 | 2.4±2.1 | 0.8±0.8 |
| **Homoterpene** |  |  |  |  |
| (*E*)-DMNT | 0.0±0.0 | 0.0±0.0 | 1.2±1.2 | 6.2±4.1 |
| **Sesquiterpenes** |  |  |  |  |
| β-Sesquiphellandrene | 6.9±4.1 | 20.1±7.1 | 5.9±2.8 | 20.3±1.3 |
| Sesquiterpene RI 1376 | 0.0±0.0 | 2.2±0.9 | 0.6±0.4 | 2.5±0.2 |
| Sesquiterpene RI 1386 | 0.3±0.3 | 1.1±0.7 | 0.3±0.3 | 1.2±0.1 |
| Sesquiterpene RI 1416 | 0.0±0.0 | 1.2±0.7 | 1.9±1.4 | 2.4±0.3 |
| Caryophyllene | 0.0±0.0 | 0.2±0.2 | 0.6±0.4 | 2.0±0.9 |
| Dehydroaromadendrene | 1.0±1.0 | 5.5±2.3 | 1.4±0.8 | 5.4±0.5 |
| Sesquiterpene RI 1533 | 2.4±1.2 | 7.5±3.3 | 2.0±1.3 | 7.2±0.7 |
| **GLVs** |  |  |  |  |
| (*E*)-3-Hexenal | 5.0±5.0 | 16.2±11.9 | 20.2±15.2 | 48.6±28.3 |
| Hexanal | 7.4±4.8 | 3.7±3.0 | 30.8±15.9 | 20.1±8.3 |
| (*Z*)-2-Hexenal | 4.0±2.2 | 9.3±5.4 | 29.5±15.3 | 43.3±17.5 |
| (*E*)-3-Hexenol | 3.8±3.8 | 25.6±14.9 | 23.5±8.7 | 34.6±15.6 |
| (*E*)-3-Hexenyl acetate | 76.4±54.6 | 217.6±157.2 | 129.2±62.9 | 335±196.3 |
| Hexyl acetate | 0.0±0.0 | 0.0±0.0 | 1.2±0.7 | 0.0±0.0 |
| **N-/or S-containing compounds** |  |  |  |  |
| Methyl sulfone | 9.3±5.2 | 0.3±0.3 | 1.5±1.1 | 0.0±0.0 |
| ***Raphanus sativus*** | **n=5** | **n=4** | **n=5** | **n=4** |
| **Monoterpenes** |  |  |  |  |
| β-Pinene | 0.0±0.0 | 2.0±0.9 | 2.6±2.6 | 2.2±1.0 |
| Δ-3-Carene | 0.0±0.0 | 0.0±0.0 | 0.2±0.1 | 0.6±0.3 |
| Limonene | 0.2±0.2 | 0.7±0.7 | 0.0±0.0 | 1.1±0.5 |
| (*Z*)-β-Ocimene | 2.9±0.6 | 0.4±0.4 | 6.3±0.9 | 6.3±0.9 |
| **Homoterpene** |  |  |  |  |
| (*E*)-DMNT | 0.0±0.0 | 0.0±0.0 | 8.3±3.1 | 36.5±9.5 |
| **Sesquiterpenes** |  |  |  |  |
| Caryophyllene | 1.9±0.7 | 2.5±0.9 | 7.3±3.7 | 2.7±2.7 |
| **GLVs** |  |  |  |  |
| (*E*)-3-Hexenal | 16.2±9.9 | 5.1±5.1 | 23.5±21.0 | 11.8±7.2 |
| Hexanal | 18.7±11.2 | 42.5±20.1 | 33.0±7.6 | 37.5±13.1 |
| (*Z*)-2-Hexenal | 13.1±8.5 | 27.1±13.5 | 22.5±9.2 | 14.5±5.0 |
| (*E*)-3-Hexenol | 18.8±9.2 | 36.6±24.9 | 23.5±7.5 | 27.4±10.1 |
| (*E*)-3-Hexenyl acetate | 280.7±111.8 | 85.3±17.9 | 105.9±39.8 | 226.3±49.1 |
| (*E*)-Hexenyl valerate | 0.4±0.4 | 0.0±0.0 | 0.3±0.3 | 0.0±0.0 |
| **N-/or S-containing compounds** |  |  |  |  |
| Methyl isothiocyanate | 7.0±2.3 | 4.0±2.2 | 5.7±0.8 | 4.9±3.3 |

**Online resource 3.** Partial Least Squares – Discriminant Analysis (PLS-DA) based on emissions rates of the wild plant species exposed to ambient ozone 15ppb (AMB); ambient ozone and *Plutella xylostella* larvae feeding (AMBHERB); 80 ppb ozone (O3); and 80 ppb ozone and *Plutella xylostella* larvae feeding (O3HERB). a) *Barbarea vulgaris*, b) *Brassica nigra*, c) *Sinapis arvensis* and d) *Raphanus raphanistrum*. The NMC score represents the percentage of misclassifications calculated for each PLS-DA analysis.

**Online resource 4.** Partial Least Squares – Discriminant Analysis (PLS-DA) based on emissions rates of the cultivated plant species exposed to ambient ozone 15ppb (AMB); ambient ozone and *Plutella xylostella* larvae feeding (AMBHERB); 80 ppb ozone (O3); and 80 ppb ozone and *Plutella xylostella* larvae feeding (O3HERB). a) *Brassica juncea*, b) *Brassica napus*, c) *Sinapis alba* and d) *Raphanus sativus.* The NMC score represents the percentage of misclassifications calculated for each PLS-DA analysis.
